# Incubation Period of Hantavirus Cardiopulmonary Syndrome

**DOI:** 10.3201/eid1208.051127

**Published:** 2006-08

**Authors:** Pablo A. Vial, Francisca Valdivieso, Gregory Mertz, Constanza Castillo, Edith Belmar, Iris Delgado, Mauricio Tapia, Marcela Ferrés

**Affiliations:** * Universidad del Desarrollo, Santiago, Chile;; † University of New Mexico School of Medicine, Albuquerque, New Mexico, USA;; ‡ Universidad de la Frontera, Temuco, Chile;; § Hospital Regional de Coyhaique, Coyhaique, Chile;; ¶ Pontifica Universidad Católica, Santiago, Chile

**Keywords:** Hantavirus, incubation period, Andes virus, dispatch

## Abstract

The potential incubation period from exposure to onset of symptoms was 7–39 days (median 18 days) in 20 patients with a defined period of exposure to Andes virus in a high-risk area. This period was 14–32 days (median 18 days) in 11 patients with exposure for <48 hours.

Hantaviruses are RNA viruses that are harbored by specific rodent species and transmitted to humans by inhalation of virus-contaminated rodent feces, urine, and saliva (*1*). Human hantavirus syndromes include hemorrhagic fever with renal syndrome (HFRS) and hantavirus cardiopulmonary syndrome (HCPS) (*2*). The latter is also known as hantavirus pulmonary syndrome, but we prefer HCPS because most deaths result from cardiogenic shock (*3**,**4*).

Although HCPS is a serious problem in North and Central America, more cases of HCPS and deaths from this disease occur in South America; in Chile, 469 cases have been reported through March 2, 2006, with a case-fatality rate of 36% (*5**,**6*). Both Sin Nombre virus (SNV), the primary cause of HCPS in North America, and Andes virus, the cause of HCPS in Chile and most cases in Argentina, cause a severe form of HCPS. However, Andes virus is unique among hantaviruses in that it can be transmitted from person to person (*7*).

Human contact with *Oligoryzomys longicaudatus* (rice rat or colilargo), the reservoir of Andes virus, occurs in rural areas in central and southern Chile (from 28°S to 51°S). In Chile, 70% of the patients have a history of occupational or peridomestic exposure to rodents or peridomestic exposure to a human with HCPS; in 20% to 35%, exposure is limited to visiting high-risk areas for recreational purposes (*8*).

The incubation period for HCPS caused by Andes virus has not been reported. The incubation period for HCPS caused by SNV has been reported to be 9–33 days (*9*). The incubation period for HFRS has been estimated to be 1–6 weeks (*10**,**11*) but was reported as 11–23 days after intramuscular or intravenous challenge in volunteers (*12*).

## The Study

To define the incubation period for Andes virus infection, we identified 20 patients with a well-defined period of exposure to a high-risk area among 106 persons with HCPS enrolled in research protocols (treatment interventions, contact studies, quantitative viremia during HCPS) or interviewed by 1 of the authors. Nineteen of 20 were residents of Santiago or other urban areas who traveled to a high-risk area for recreational purposes. In each case, the person resided in an urban area without Andes virus–infected rodents and rodent-to-human transmission and then traveled for a defined period to a high-risk area where rodent-to-human transmission has occurred and where Andes virus–infected rodents were found (*13*). Nineteen patients reported a variety of risky activities, such as entering or cleaning previously unused cabins or houses, camping, or clearing land. The other patient (no. 11) was a biologist who was bitten on the finger by a rodent, which he identified as *O*. *longicaudatus* that he had trapped in a rural area.

The exposure period was defined as the number of days from arrival to departure in a high-risk area. The maximum incubation period was the time from arrival at the high-risk area to the onset of symptoms, and the minimum incubation was the time from departure from the high-risk area to onset of symptoms. The prodrome was defined as the period from the onset of fever or other constitutional symptoms until the onset of the cardiopulmonary phase and hospitalization.

Confirmation of HCPS was based on the clinical syndrome with laboratory confirmation by >1 of the following tests: ELISA for immunoglobulin G (IgG) and IgM antibody for hantavirus, a focus reduction assay for neutralizing antibody to Andes virus, and an RNA reverse transcription (RT)-PCR for Andes virus. Laboratory confirmation was by IgG and IgM ELISA in 8 patients; IgG and IgM ELISA plus Andes virus neutralizing antibody in 3 patients; IgG and IgM ELISA, Andes virus neutralizing antibody, and RT-PCR in 7 patients; IgG and IgM ELISA plus RT-PCR in 1 patient; and RT-PCR in 1 patient. Patients had a mean age of 30.5 years (range 2–68 years); 65% were male. The clinical course was characterized as severe (respiratory failure and shock) in 14, moderate (respiratory failure without shock) in 1, and mild (respiratory failure without requiring mechanical ventilation) in 5. Four of 20 patients died.

The potential maximum incubation period for all 20 patients was 11–39 days, and the potential minimum incubation period was 7–32 days. The median incubation period for all 20 patients was 18 days (range 7–39 days) (Figure). Among the 11 patients (nos. 10–20) with exposure <48 hours, the potential incubation period was 14–32 days (median 18 days). The incubation periods of patients with mild cases (range 12–34 days) did not differ from those of patients with moderate and severe cases (7–39 days).

**Figure:**
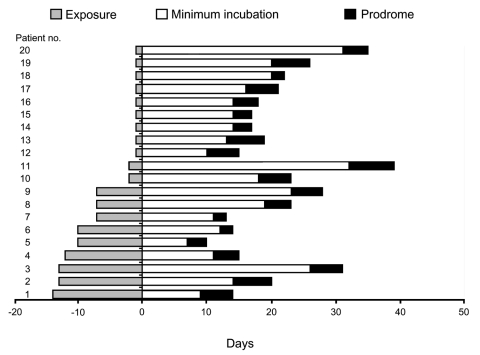
Incubation period for 20 patients in Chile in whom hantavirus cardiopulmonary syndrome caused by Andes virus developed after various periods of exposure. All patients progressed to the cardiopulmonary phase and were hospitalized at the end of the prodrome.

## Conclusions

Our study is the first to determine the incubation period for Andes virus infection. It provides the most complete evaluation of the incubation period for rodent-to-human transmission for the New World hantaviruses, including Andes virus and SNV. Young et al. reported 11 patients with SNV infection with well-defined exposure to rodents (*9*). However, only maximum or minimum incubation periods could be determined for 4 patients. In the 7 patients for whom both minimum and maximum incubation periods could be calculated, the incubation period had a range of 9 to 33 days. St Jeor reported SNV infection in 2 children hospitalized 3 weeks after they were bitten by a mouse (*14*), but the time between the bite and the onset of symptoms was not reported.

Human-to-human transmission of Andes virus infection has been reported in Argentina (*7**,**15*) and Chile where human-to-human transmission is strongly suggested in household clusters of HCPS cases (M. Ferrés, pers. comm.). In the 1996 outbreak in Argentina, both epidemiologic and molecular evidence supported person-to-person transmission (*7**,**15*). The time between disease onset in 14 cases of person-to-person transmission among 16 patients with HCPS was 4–28 days. However, these intervals should be interpreted with caution. They are based on the mode of transmission considered most likely by Wells et al. (*15*), but there were multiple cases in which patients had contact with >1 potential source patient. Furthermore, these were intervals between onset of symptoms in the proposed source and in subsequent patients, and with 4 exceptions, were not calculations of an incubation period based on defined periods of exposure to the proposed source patient. The duration of exposure to source patients was reported for only 4 case-patients, including 3 occupants of a car in whom symptoms developed at 11, 15, and 29 days, respectively, after a daylong car trip with an index patient who was symptomatic. The shortest interval of 4 days was for a patient who had close contact with another patient 10 days before symptoms developed. If this patient is considered to be a more likely source, as it was by Wells et al., the range would be 10–28 days.

In summary, our data for 11 patients in whom exposure was limited to <48 hours showed a potential incubation period of 14 to 32 days and a median of 18 days. Inclusion of patients with exposure periods <14 days provided a potential incubation period of 7 to 39 days. These data provide the most complete evaluation of the incubation period for HCPS caused by Andes or SNV and are consistent with available data for the incubation period for HFRS (*7**,**9**–**12**,**14**,**15*).
